# Hydrophobia1Read before the Virginia State Veterinary Medical Association, Charlottesville, Va., January 5, 1895.

**Published:** 1895-03

**Authors:** J. H. Adamson


### HYDROPHOBIA.[1]

BY J. H. ADAMSON, D.V.S.

GENTLEMEN: The subject of my essay, "Hydrophobia," or "Rabies," is chosen simply to invoke, stimulate, and invite discussion in this meeting of veterinarians and members of the medical profession, if any are present with us to-day.

HISTORY. Plutarch asserts that, according to Athenordorus, the disease was first discovered in mankind in the days of Asclepiades, the descendants of the God of Medicine (Esculapius), by his sons, Podalirius and Macheon. Hydrophobia is also alluded to in the works of Aristotle, Xenophon, Virgil, Horace, Ovid, Celsus, Galen, and subsequently investigations were published by Boerhaave, Van Swieten, John Hunter, Majendie, Breschet, Virchow, Reder, Hertwig, Pasteur, Klebbs, Koch, Sternberg, Williams, Fleuring, Meynell, and many other notable men of our day.

In 1271 wolves were known to be affected by rabies in Franconia. In 1590 rabies prevailed in Spain, also in the same year

[1] Read before the Virginia State Veterinary Medical Association, Charlottesville, Va., January 5, 1895.

at Monthelliard. During the early part of the century rabies was very prevalent both in this country and in Europe, and there were serious outbreaks in England as late as 1869, 1876 and 1878. It has occurred in every State in this Union at some time or another during the present century, but, fortunately, never to any alarming extent. The disease is hardly known in South America and Egypt. Syria and Greenland are exempt.

CAUSE. Hydrophobia is a specific blood-disease, due to some germ that has not, as yet, been discovered. It has not been isolated or cultivated, but inoculation experiments have demonstrated its presence in the blood of the affected animals. It is known beyond doubt that a specific virus is developed in the saliva of all affected animals, and implanted through a wound or abrasion in the epidermis or mucous membrane conveys the disease to man and other animals.

That its propagation is generally due to inoculation cannot be disputed, but its apparent sources of spontaneous origin, how produced, or where obtained by the victim are circumstances which, as yet, are hidden in obscurity. The influence of climate, food, hunger, thirst, or domestication do not produce rabies. The non-gratification of sexual desire, as well as severe pain, is considered by some authorities to be at least an exciting cause of this disease. Solitary confinement has also been advanced as an exciting cause. The idea that hot weather is very productive of rabies is disproved by the tabulated report of Prof. St. Cyr, of the Lyons Veterinary Institute. It plainly states that rabies is even more prevalent during the temperate months than those of extreme heat or cold.

According to M. André, the hottest and coldest months furnish the fewest cases.

PATHOLOGY AND SYMPTOMS OF RABIES. Three circumstances in the pathology of rabies are worthy of notice, namely:

1. That the period of latency, or incubation, after inoculation is very indefinite, ranging from fourteen days to several years, and that it varies in different animals.

2. That inoculation does not always produce the disease, as one-fourth of inoculated animals escape.

3. That the disease is transmissible to man, mammifera, and birds by the usual media at a later period than twenty-four hours after the death of a rabid animal, or after cadaveric rigidity (rigor mortis) has completely invaded the body.

The symptoms of canine madness are very much the same in all cases, though varying somewhat in their manifestations. Generally the mad dog shows a warning of latent disease developing, by a change in manner, for several days before it breaks forth in severity. The countenance becomes anxious and appealing, the eyes become bloodshot and singularly bright and sparkling, while a slight tendency to distorted vision or squinting is apparent. Such alteration in the usual appearance of the eyes and countenance is generally followed by mischievous propensities possessed by the animal in health. An array of symptoms now begin to encroach quickly one upon another until a lamentable spectacle is presented. The appetite suddenly becomes depraved, lapping of urine, devouring excrement, wood, stones, and all sorts of foreign bodies. This symptom of depravity alone is characteristic of hydrophobia, and is amply sufficient to justify any man in making his diagnosis, according to Prof. Lagarris, of the Pasteur Institute in Chicago. The next important symptom is one which is also considered most demonstrative, viz., an insatiable thirst, a pitiable craving for water or any other liquid, which is swallowed eagerly, the animal pushing his head, over muzzle, up to the eyes in the vessel containing the liquid, in his frantic attempts to quench his burning thirst. He will wallow through and even swim across ponds or streams of water, throughout the whole course of attack.

The animal now becomes exceedingly restless and irritable; he wanders aimlessly around, never walking in a straight line, but in zig-zag fashion; will crowd behind chairs or other articles of furniture, and attempt to pass between them and the wall, knocking them over in his hurry. If he is confined by a chain, he attempts to gnaw and chew it to pieces; if by a door, he vents his fury on that. In this state he knows not the sensation of ordinary pain, as he will bite a red-hot poker if presented to him exactly as if it were a cold one. In drinking he will usually upset the stock of fluid in his great hurry to imbibe. The attempt to swallow produces violent paroxysms of the larynx and pharynx, until finally the unhappy sufferer ceases from sheer dread to quench the thirst which torments him. The hysterical bark suddenly changes from an angry tone to one of joy and ecstasy; then a whine, as if asking for something; again, a melancholy, dismal howl, as if lost or that some approaching trouble was at hand. Then an interval of complete naturalness, followed in a few minutes by trismatic convulsions, which, however, soon disappears, and the howling commences again.

If allowed liberty, the rabid dog always seeks seclusion as the disease advances. He will leave his home and go off in a perfectly straight line along the street. The gait is peculiar and characteristic of the rapid dog, and nothing else. It may be described as a shuffling motion, a mixture between a pacing and trotting movement, commonly known as the fox-trot. He holds his head in a perfectly straight line with the body, and looks neither to the right nor to the left. When compelled to alter his course he always turns at right angles. He will not go one inch out of his way to do any mischief or to avoid danger, and will often pass through crowds of people without ever thinking of biting them; and even if pursued by cries and hooting he takes no notice until he is hurt bodily; then he will wreak his vengeance on the offender or anything else that comes his way. More frequently he will endeavor to escape his assailants and reach solitude, where, once by himself, he will snap at imaginary objects, and will only attack real ones if placed in his way. This snapping at the empty air is also very characteristic.

The prevailing desire to roam and wander away seems to me to be an instinctive attempt to get rid of the disease by muscular action, as rabies is spontaneous only in non-perspiring animals.

When the disease first declares itself, if it has been produced by inoculation, it not infrequently happens that the wound, which had rapidly and entirely healed after the bite, begins to exhibit evidence of irritation or inflammatory action, tingling and itching severely, which causes the animal to gnaw and bite it, without even feeling the slightest pain therefrom, as though there was a morbid sensation of numbness in that region.

In man, and other animals than the dog, the breathing becomes more laborious and jerking, accompanied by peculiar sonorous expirations which often suggest to observers the notion that the sufferer "barks like a dog." During the latter stages there is always a viscid secretion in the mouth, which is ropy, thick, and gluey. There is never great fever, but the bowels are always constipated, and there is a diminished flow of urine. After two or three days of suffering of the most terrible description the animal succumbs, death taking place either from a paroxysm of choking or in a tranquil manner from nervous exhaustion, all the symptoms having abated and the power of swallowing having returned shortly before death. The duration of the disease is generally from two to five days.

Treatment. Medicinal treatment of rabies is of no avail whatever. An antidote for the ptomaine has not yet been discovered. Pasteur says that the blood-serum of a recovered case after undergoing certain preparation and treatment, at present only known to his laboratory, is a speedy and sure cure. However, the world has not as yet recognized the fact.

Medicines cannot be administered by the mouth, owing to the impossibility of swallowing, the distress occasioned by the effort to do so, and the great risk of being bitten by the sufferer. Therefore, anything given must be per rectum, hypodermatically, or by inhalation. The most potent agents are the bromides, opium, its alkaloids, curara, chloral, chloroform, and hydrocyanic acid. The vapor bath is also recommended; but in this, also, great risks are run. Prophylactic treatment is the only rightful course to pursue, and of most avail. When an animal or man is first bitten by a rabid or apparently healthy dog, endeavor to prevent the absorption of the virus into the system. This may be accomplished by complete excision of the part involved in the bite, or by sucking the wound by a cupping glass, or even by the mouth if practicable, after which the part ought to be thoroughly cauterized by nitrate of silver, caustic potash, nitric acid, or, better still, actual cautery. Then place the animal in confinement to await developments. One month is considered a long enough period.

Do not kill the dog, as it prevents the persons bitten from knowing whether hydrophobia will develop or not. If it does not mature, the persons bitten are perfectly safe, and may sleep easy.

The reprehensible practice of muzzling dogs in hot weather ought to be prevented by law. No animal suffers more intensely from thirst than the dog, and it is gross ignorance on the part of the laity who practice it, and it is gross cruelty to the animal, while it serves no good end whatever.

Pathological anatomy. Post-mortem examinations have thrown much new light upon this malady, and much special attention has been given by certain eminent pathologists to this work. The chief morbid changes which are described are evidences of congestion and inflammatory action in certain portions of the brain and spinal cord, and most particularly in the locality known as the "respiratory centre" of the medulla-oblongata, where the accumulation of "leucocytes" around the small bloodvessels and in the surrounding nervous substances are a prominent phenomenon. Similar changes are also found in the salivary glands.

The eighth pair of nerves, which are largely concerned in the processes of respiration and deglutition, are congested in a marked degree, and it is upon this portion of the nervous system that the poison or ptomaine most powerfully exerts its specific action. But that the whole great nerve centres, viz., the brain and spinal cord, as a whole, are profoundly affected, is manifest in the tendency to general convulsion, the remarkable hyperæsthesia, and the mental perturbation of the victim.

I could write more on the latest experiments on rabies and remarks on post-mortem by many able men, also much on treatment by our recent bacteriologists, such as Sternberg, Gmelin, Lehman, Marchand, and others; but of this you can read at your leisure. I will, however, quote one master mind at the risk of him quoting Pasteur:

"From the symptoms observed during life the conclusion naturally suggests itself that the brain and its membranes are the seat of organic lesions. Indeed, the specific action of the poison appears to be exercised, particularly in the first instance, upon the medulla oblongata and the par vagum, the branches of which seem to lose their natural properties; hence the difficulty in swallowing, breathing, the depraved appetite, alteration of voice, and its entire loss in the dumb form, as well as the convulsions of respiratory muscles, are all due to the derangement of this nerve; and as the nervous system becomes more and more deranged complete paralysis of the respiratory muscles occurs, and the animal dies of asphyxia.

"The principal post-mortem appearances are œdema or congestion, sometimes in patches, of the brain and spinal cord, particularly at the base and plexus choroids, effusion into the arachnoideal space, cerebral ventricles, and the cerebro-spinal substance, and softening of the membranes. On the lower surface of the medulla-oblongata, at the margin of the seventh, eighth, and ninth pairs of nerves the membranes are generally thickened, softened, and clotted together. The liver, spleen, kidneys, and muscular system are congested. The bladder is empty and its mucous membrane covered with petechiæ. The lungs are engorged with blood. The blood in the vessels is imperfectly coagulated, often black and tarry, sometimes bright and red in appearance.

"The mucous membrane of the pharynx, œsophagus, stomach, and bowels are either greatly congested or diffusely inflamed. Patches of extravasation are particularly met with on the gastric mucous membrane, which accounts for the hemorrhagic vomiting which is often witnessed during the illness. The contents of the stomach consists of all sorts of foreign substances. The tongue is often wounded by the teeth, its papillæ congested, and the salivary glands enlarged and vascular. In 'dumb madness' the congestions of the upper part of the respiratory tract are developed to a greater degree than in any other form of this disease."

REMARKS. John Hunter says: "Individual susceptibility must be taken into account, as it is undeniable that many persons in whom the virus of rabies has been inoculated escape hydrophobia. One instance that came under my notice, in which twenty-one persons were bitten by a rabid dog (proven so beyond doubt) only one died subsequently from hydrophobia, and comparisons by authentic authorities go to show that not one-third of those persons bitten by rabid animals die of rabies. A person bitten through his clothing is comparatively safe. Only 1 per cent. die of rabies.

"It is supposed by some that the bite of an angry dog may produce rabies, and all the more so if the animal should develop hydrophobia years after. The non-rabid animal, however enraged, cannot give rise to hydrophobia by his bite. Many persons die from mental derangement (delirium) produced by the fear of the consequences following a bite."

Treviranus found that all saliva reddened by the addition of sesquichloride of iron; Leopold Gmelin discovered that this was caused by a sulpho-cyanate which is present in saliva. Poisoning from cyanic acid resembles slightly the latter stages of rabies.

The following paragraph appeared in the London *Herald of Health*, 1894:

"It has been stated in this journal on previous occasions that the virus of rabies can be removed from perspiring animals by means of vapor baths. In non-perspiring animals, such as the dog, wolf, cat, and other flesh-eating animals, it is considered to be invariably fatal in its effects. Such animals should not be kept in captivity unless kept hygienically clean, otherwise they are dangerous amidst human life. Any poison or virus that enters the body permeates the whole system. The pores of the skin allow much of its escape. In the non-perspiring animals this cannot occur, hence great muscular action during rabies in the dog. Dr. Buisson, of Paris, was inoculated with the virus of rabies from a patient, and the symptoms of the disease having become extreme, he resolved to die in a vapor bath of 127° F., but instead of dying he was cured. This was twenty-nine years ago, and Dr. Buisson is living to-day, so there is no likelihood of the disease lying latent. It was not suppressed; it was entirely removed, and that beyond a doubt. Dr. Buisson has compiled a work on this treatment, and quotes many instances of recovery from rabies by vapor treatment that have occurred in his practice."

M. Pasteur wrote to a lady in Glasgow, Scotland, last year: "The bite of a dog is only dangerous when he is suffering from rabies. If there is any doubt about the animal's disease (if diseased) observe the following advice:

"Place the animal where it can do no further harm; have it examined by a qualified veterinarian; he will soon discover the characteristic symptoms of rabies. If suffering from rabies, he will surely die in eight days. If, at the end of two weeks, no symptoms of rabies have developed, the bite cannot cause hydrophobia, and there is no reason that the animal should be destroyed."

